# Trends and Classification of Artificial Intelligence Models Utilized in Dentistry: A Bibliometric Study

**DOI:** 10.7759/cureus.81836

**Published:** 2025-04-07

**Authors:** Mohammadjavad Shirani

**Affiliations:** 1 Department of Restorative Dentistry, Kornberg School of Dentistry, Temple University, Philadelphia, USA

**Keywords:** artificial intelligence, deep learning, dental treatment, dentistry, machine learning

## Abstract

This bibliometric study introduces a novel approach to assessing the application of artificial intelligence (AI) in dentistry. It analyzes trends in AI utilization across dental disciplines, treatment stages, data modalities, subsets, models, and tasks and proposes a comprehensive classification framework for AI applications in dentistry. A systematic search in the Web of Science Core Collection on December 1, 2024, using AI- and dentistry-related keywords identified original and review articles employing true AI. Data on publication details, study types, dental disciplines, treatment stages, AI subsets, models, data modalities, and tasks were extracted and analyzed using VOSviewer (Leiden University, Leiden, Netherlands) and Microsoft Excel (Microsoft Corp., Redmond, WA). Trend analysis and forecasting methods were applied to identify future research directions. Of 2,810 records, 1,368 studies met the inclusion criteria, revealing a continuous rise in AI-related dental research. While most studies focused on diagnostic applications and the orthodontics discipline, the highest recent growth was seen in treatment planning and research and education applications. Hybrid AI models and natural language processing (NLP) experienced significant increases in adoption. The most common AI tasks were classification, detection, and segmentation, although notable growth occurred in generation, data integration, and decision support. The classification framework for AI in dentistry is presented. Text-based data have shown the greatest growth among data modalities, alongside an increased use of sensor and signal data. Future research should prioritize developing NLP and hybrid AI models, conducting original studies in research and education and treatment planning, and undertaking systematic reviews focused on the diagnosis stage of prosthodontics and endodontics.

## Introduction and background

Artificial intelligence (AI), a branch of computer science, possesses the ability to perform diverse tasks such as pattern recognition, reasoning, learning, problem-solving, perception, and natural language understanding [1,2]. The AI systems employed in various fields, including dentistry, range from basic rule-based systems and traditional machine learning (TML) to advanced deep learning (DL) and hybrid models. Both TML and DL are subsets of a broader category known as machine learning (ML). TML involves systems that learn from smaller datasets using algorithms, such as random forests (RF) and decision trees (DT), typically requiring manual feature extraction. DL utilizes neural networks with multiple hidden layers that automatically learn more complicated features from raw large datasets [3-5].

ML involves systems that learn from data and make decisions or predictions without explicit programming [6]. DL, an advanced type of ML, employs neural networks comprising multiple interconnected layers: one input layer, some hidden layers, and one output layer. This architecture enables AI systems to autonomously identify and process complex patterns in large datasets [3-6]. The recent generation of AI integrates specialized domain knowledge with advanced computational techniques, resulting in hybrid models (systems combining multiple AI approaches to enhance performance and adaptability), generative systems, explainable AI (systems that provide transparent and interpretable reasoning behind their predictions), and foundation models (large-scale pre-trained models adaptable to a wide range of tasks) [6,7].

The application of AI in dentistry has gained significant attention, necessitating that researchers and clinicians stay informed about emerging AI models and their capabilities [8-12]. As AI adoption in dental practice expands, understanding its advancements is crucial for optimizing its integration into clinical workflows. Given the growing AI-related dental research, it is essential to identify knowledge gaps and guide future investigations [9-14].

A structured classification of AI systems in dentistry, coupled with an analysis of trends and future projections, can benefit both researchers and practitioners. Bibliometric studies provide a quantitative approach to evaluating scientific output and identifying patterns, trends, and thematic developments within a field [15-18]. While some bibliometric analyses have examined AI applications in dentistry, they have largely focused on publication metrics and general trends [18,19].

To date, no bibliometric study has systematically explored AI applications across the stages of dental treatment. Although AI classifications have been discussed in broader computer science literature [4,20] and some dental studies [10,21], a comprehensive, structured classification tailored to AI systems used specifically in dentistry is lacking. This study proposes a novel framework to fill that gap and facilitate better understanding across disciplines.

This study seeks to address this gap by employing a novel approach to systematically analyze trends and forecast future directions in AI applications across the four stages of dental treatment. Additionally, it provides a detailed classification of AI models employed in dentistry, offering statistical insights into their development and adoption.

## Review

Methods

This bibliometric study addresses two key research questions: (1) What are the trends in AI applications across dental disciplines and the four stages of dental treatment, examination and diagnosis, treatment planning, operative phase, and follow-up? (2) How have different AI subsets, models, data modalities, and tasks evolved in dental research? The study follows a structured bibliometric framework and adheres to established guidelines for reporting bibliometric reviews in biomedical literature [15,16].

Search Strategy

A comprehensive search query combining AI and dentistry-related keywords was developed. The search strategy was applied to the title and abstract fields in the Web of Science Core Collection on December 1, 2024: ((“Artificial Intelligence” OR AI-based OR “Computational Intelligence” OR “Machine Learning” OR “Deep Learning” OR “Neural Network” OR “Expert System” OR Robot* OR “Natural Language Processing”) AND (Dentistry OR “Dental Treatment” OR Tooth OR Teeth OR “Oral Medicine” OR “Oral Diagnosis” OR “Oral Pathology” OR “Oral Lesion” OR “Oral Radiology” OR “Periapical Lesion” OR Endodontics OR “Carious Lesion” OR “Restorative Dentistry” OR Prosthodontics OR “Dental Implant” OR “Oral Implantology” OR Periodontics OR Periodontology OR “Oral and Maxillofacial Surgery” OR Orofacial OR Orthodontics OR Pedodontics OR “Forensic Dentistry” OR “Oral Public Health”)).

All retrieved publications were exported into the EndNote software (Clarivate, London, United Kingdom) for management. This study included only English-language publications of review studies and original articles as primary studies (interventional, observational, and in vitro) and proof-of-concept studies (algorithm development, technology prototypes, and pilot studies) focusing on the application of true AI in dentistry.

For the purposes of this study, true AI was defined as a model with the ability to learn, recognize patterns, and reason, encompassing tools from simple rule-based systems to advanced deep learning techniques [1,2]. Publications that did not meet these criteria were excluded prior to data extraction. Two independent reviewers screened the retrieved entries based on their titles, abstracts, and, if necessary, full texts. Eligible entries underwent further scrutiny by assessing their methods sections to extract the required data. When necessary, additional searches were performed to obtain supplemental information about the used AI types and models.

Data Extraction and Analysis

The following information was extracted from eligible studies and organized in an Excel (Microsoft Corp., Redmond, WA) spreadsheet: list of authors, year of publication, study title, source (journal or conference), keywords, study type, thematic focus, data modality processed by AI, subset of utilized AI, algorithm or model of AI, relevant model architecture, and AI task(s) performed in the study. The extracted data were categorized by publication year to analyze trends.

The theme of each study was categorized based on its direct involvement in one or more of the four dental treatment stages and its relevance to specific dental disciplines. Dental treatment typically consists of four main stages: (1) patient interviewing, clinical and paraclinical examinations, information gathering, diagnosis, and the creation of a problem list; (2) the development of potential treatment plans based on the problem list and collected data, followed by a discussion with the patient regarding prognoses, advantages, and disadvantages to finalize an appropriate plan; (3) the operative phase, during which the selected treatment is implemented; and (4) follow-up and maintenance to ensure the long-term success of treatment outcomes [22].

Studies were also categorized by dental discipline, including oral medicine/pathology, periodontics, endodontics, orthodontics, pediatric dentistry, oral and maxillofacial surgery, implantology, prosthodontics, operative dentistry, oral and maxillofacial radiology, forensic dentistry, oral public health, and research and education. Review studies were further categorized by their focus on specific disciplines versus general thematic attention.

Data modalities were classified into the following categories: textual, radiographic, 2D photographs, 3D scans/models/videos, and other modalities [6,23]. The types of AI were categorized into six subsets: expert systems, TML, DL, robotics, natural language processing (NLP), and hybrid models [24-27]. Details regarding the algorithms, models, architectures, and tasks performed by the AI were extracted. Based on these study findings and current AI classifications, a new classification framework for AI systems in dentistry was proposed [4,20].

The tabulated data were exported to the VOSviewer software (version 1.6.20, Leiden University, Leiden, Netherlands) to identify the most popular sources, frequently used keywords, authors with the most publications, countries with the highest number of relevant publications, and the most highly cited articles. Trends in study types, dental treatment stages, disciplines, AI subsets, models, data modalities, and tasks were analyzed based on the year of publication within the Excel software.

Trend lines were drawn for 2020-2024 for all groups within each variable to investigate trends. Considering that only the publication data from the first 11 months of 2024 was available, the actual numbers for 12 months were estimated for trend analysis to ensure consistency in the dataset. For items with the most notable characteristics or alterations, the approximate predicted values for 2025 were calculated using the appropriate forecasting methods. To ensure accurate forecasting, the selection of predictive models was based on the observed trend patterns for each variable. A linear regression analysis was performed to assess the goodness of fit (R² values).

For variables with a strong linear trend (R² > 0.9), the FORECAST.LINEAR was applied. For variables showing weaker linearity or potential non-linear trends (R² < 0.7), FORECAST.ETS was used to adjust potential non-linear fluctuations. For variables with moderate linearity (0.7 ≤ R² ≤ 0.9), the forecasting method was chosen based on visual trend analysis. For variables demonstrating exponential growth characteristics, Log-ETS was employed, using a log transformation prior to forecasting to enhance trend detection and accuracy. Irregular trends that did not fit a linear or exponential pattern were analyzed using Holt’s Double Exponential Smoothing. For variables with limited data points and high fluctuations, forecasting was deemed not applicable.

Results

Search and Screening Results

**Figure 1:**
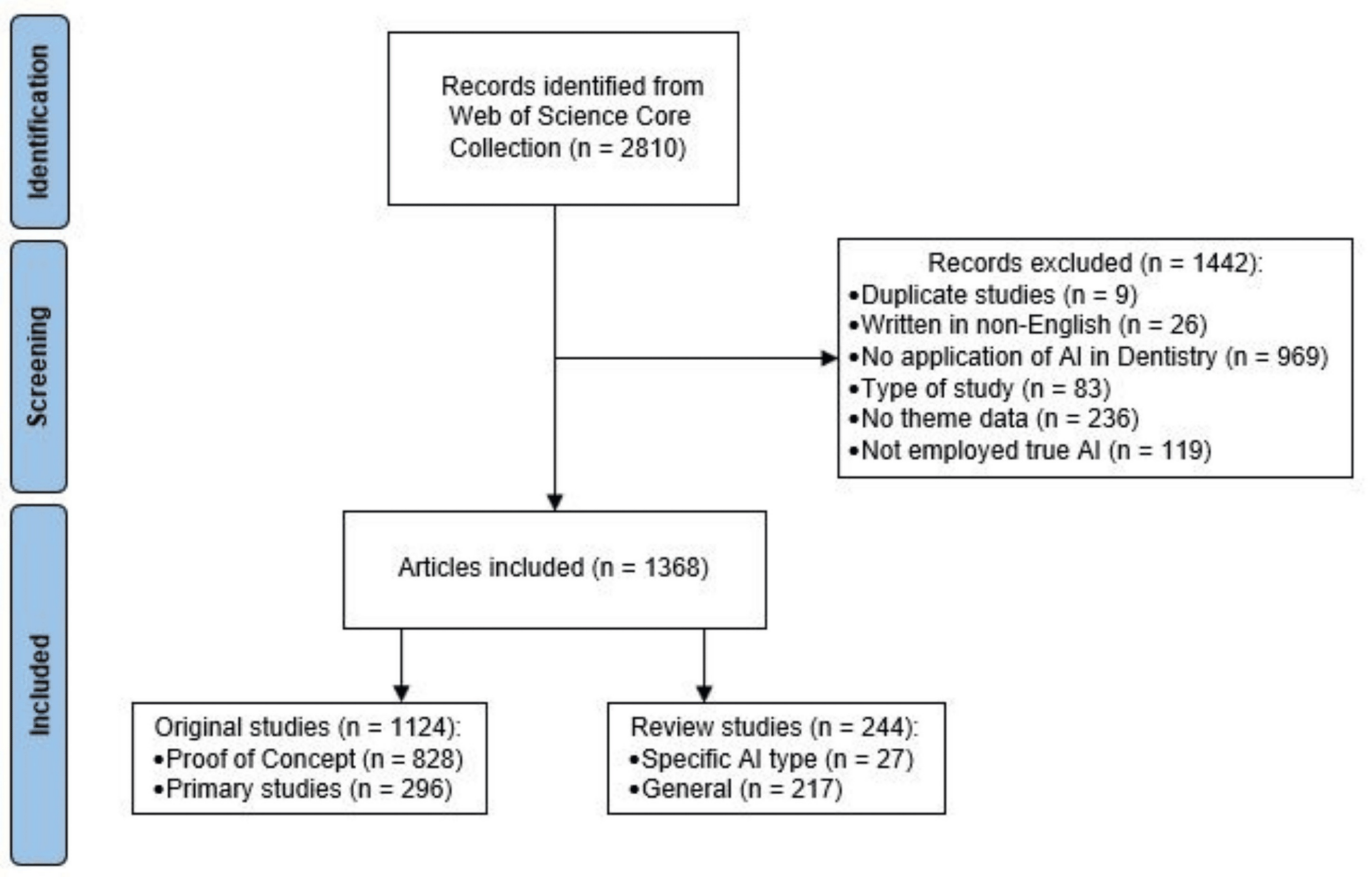
Flowchart of search results and included studies AI: artificial intelligence

Figure 1 presents the flowchart of search results and included studies. Out of 2,810 retrieved entries, 1,368 articles met the inclusion criteria. A total of 236 articles were excluded due to a lack of thematic relevance. These excluded studies primarily focused on AI in dentistry without applying AI directly within dental fields. Most of these were surveys assessing the knowledge or attitudes of dental professionals toward AI applications. Additionally, robotic studies that did not involve “true AI,” as defined in this study, were excluded.

**Figure 2:**
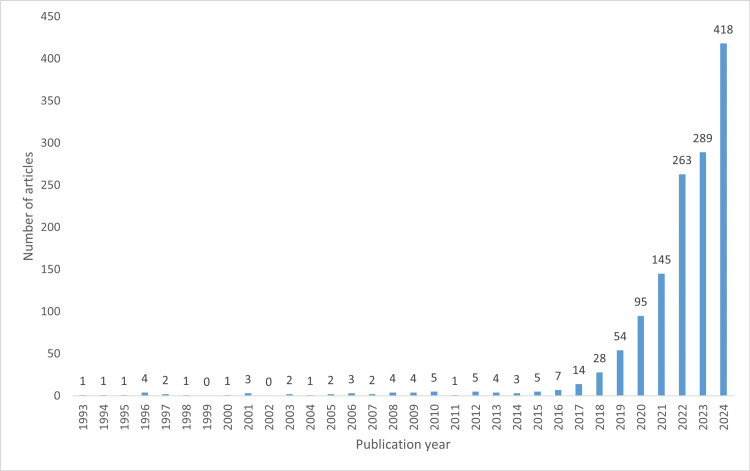
Distribution of published articles by year

**Figure 3:**
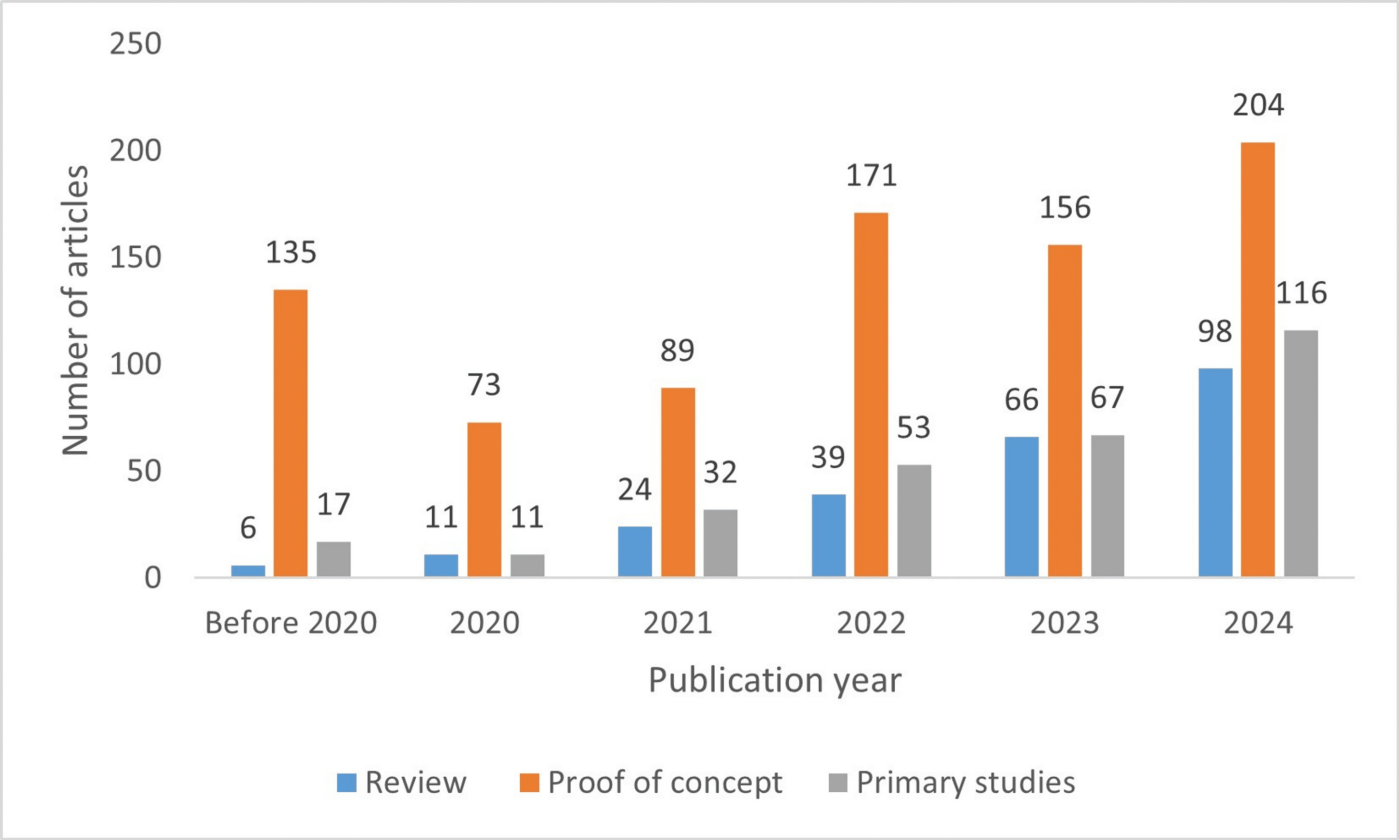
Distribution of study types across publication year groups

The distribution of published articles by year is shown in Figure 2. Due to the limited number of studies before 2020, all publications from 2019 and earlier were grouped as “before 2020” for trend investigation. Among the included articles, 244 were review studies, 828 were proof-of-concept studies, and 296 were primary studies. Figure 3 depicts the distribution of these study types across publication year groups. Notably, the number of primary and review studies in the 2023 and 2024 groups became comparable to the number of proof-of-concept studies.

Keyword, Source, Author, Country, and Citation Analysis

**Figure 4:**
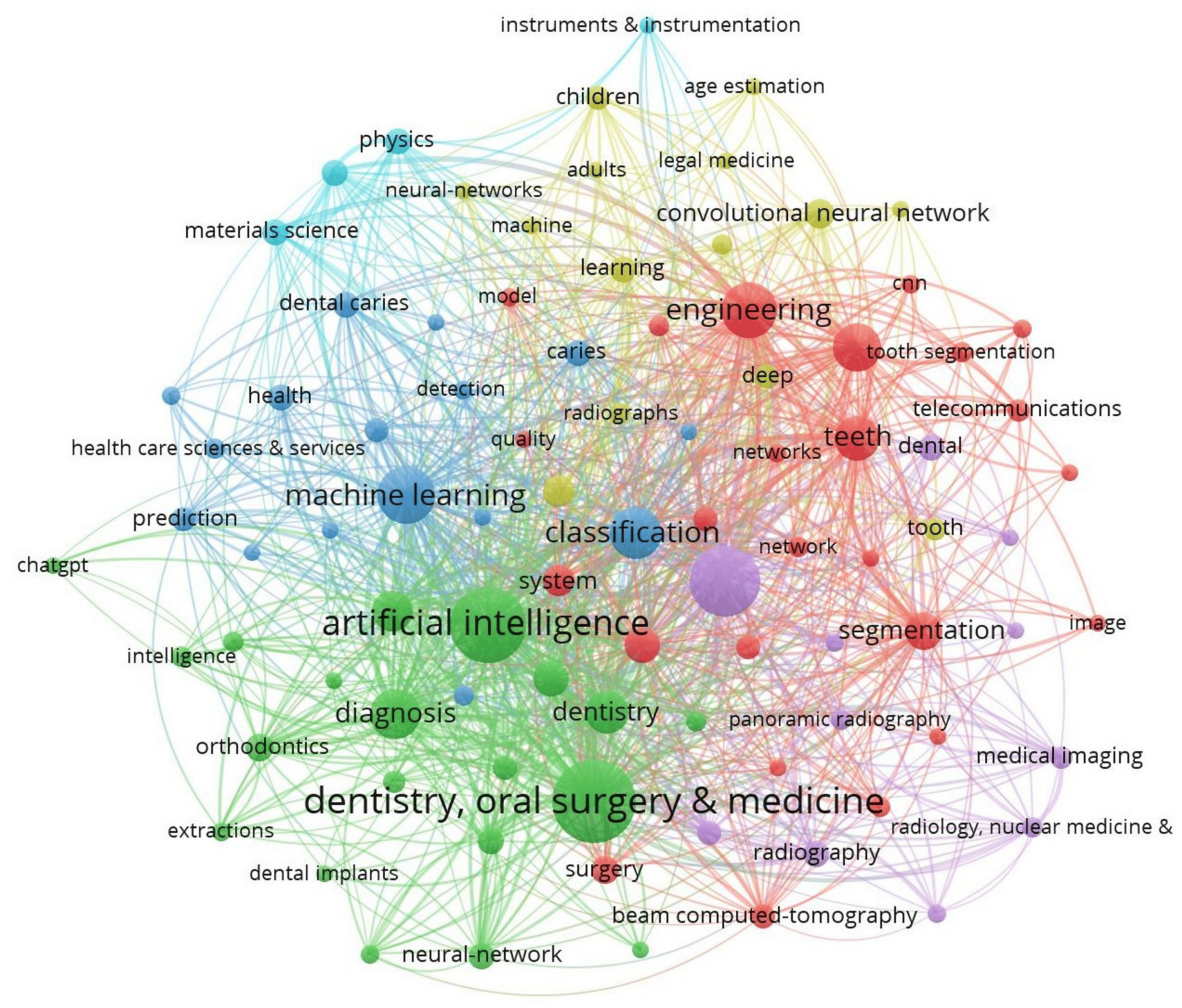
Keywords appearing 20 or more times and their collaborative relationships Larger circles represent higher frequency among the studies. The thickness of the connecting lines indicates the strength of the relationship between terms used together in a study. The same color denotes clusters with strong connections

**Figure 5:**
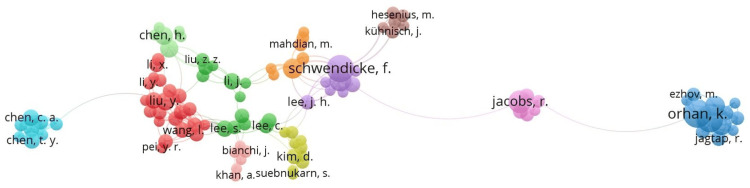
Clusters of authors with five or more publications The same color represents clusters of authors who collaborate frequently. Larger circles indicate a greater number of publications. Connecting lines show the relationships between clusters

A total of 3,911 keywords were identified across the included studies. Table 1 lists the 10 most frequent keywords and the five most common publication sources for all year groups. A temporal analysis revealed an increasing prevalence of dentistry-related keywords relative to AI-related terms. Keywords appearing 20 or more times are visualized in Figure 4, highlighting frequent terms such as “dentistry, oral surgery & medicine” and “artificial intelligence”. The journals with the highest number of published articles are the Journal of Dentistry, Scientific Reports, and BMC Oral Health. Figure 5 illustrates the 11 clusters of authors with five or more publications, underscoring the active contributors in this field.

**Table 1:** The most frequent keywords and the most common publication sources for all year groups

	Before 2020	2020	2021	2022	2023	2024
Keywords	dentistry, oral surgery & medicine; engineering; computer science; deep learning; artificial intelligence; classification; diagnosis; machine learning; neural network; segmentation	artificial intelligence; deep learning; engineering; computer science; machine learning; dentistry, oral surgery & medicine; teeth; classification; diagnosis; system	dentistry, oral surgery & medicine; deep learning; artificial intelligence; classification; machine learning; engineering; teeth; diagnosis; computer science; convolutional neural network	dentistry, oral surgery & medicine; artificial intelligence; deep learning; machine learning; classification; engineering; diagnosis; teeth; computer science; dentistry	dentistry, oral surgery & medicine; deep learning; artificial intelligence; machine learning; diagnosis; engineering; classification; general & internal medicine; computer science; dentistry	dentistry, oral surgery & medicine; artificial intelligence; deep learning; machine learning; engineering; dentistry; diagnosis; computer science; classification; general & internal medicine
Source	IEEE Access, Journal of Dental Research, Applied Sciences (Basel), Scientific Reports, and International Journal of Computer Assisted Radiology and Surgery	IEEE Access, Applied Sciences (Basel), Diagnostics, International Journal of Environmental Research and Public Health, and Journal of Clinical Medicine	Orthodontics & Craniofacial Research, Scientific Reports, Diagnostics, Journal of Clinical Medicine, and Journal of Dental Research	Scientific Reports, Diagnostics, Applied Sciences (Basel), Dentomaxillofacial Radiology, and Journal of Dentistry	Diagnostics, Cureus Journal of Medical Science, Scientific Reports, BMC Oral Health, and Applied Sciences (Basel)	Journal of Dentistry, BMC Oral Health, Scientific Reports, Cureus Journal of Medical Science, and Journal of Clinical Medicine

The highest number of publications originated from China (242 studies, 17.7%), followed by the United States (161 studies, 11.8%) and India (94 studies, 6.9%). The most cited article was “Detection and Diagnosis of Dental Caries Using a Deep Learning-Based Convolutional Neural Network Algorithm” by Lee et al., published in the Journal of Dentistry in 2018, with 439 citations. The second most cited article, with 348 citations, was “Artificial Intelligence in Dentistry: Chances and Challenges” by Schwendicke et al., published in the Journal of Dental Research in 2020. The third most cited article was “Diagnosis and Prediction of Periodontally Compromised Teeth Using a Deep Learning-Based Convolutional Neural Network Algorithm” by Lee et al., published in the Journal of Periodontal and Implant Science in 2018, which received 228 citations.

Discipline and Stage of Dental Treatment

**Figure 6:**
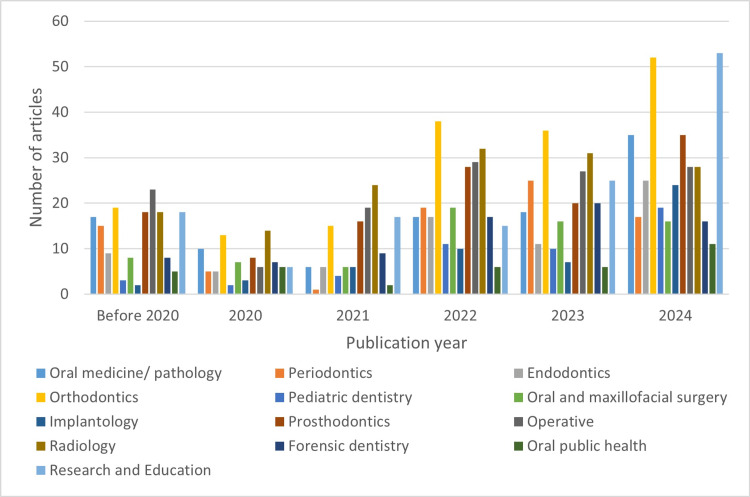
Distribution of studies for each discipline across different publication year groups

**Figure 7:**
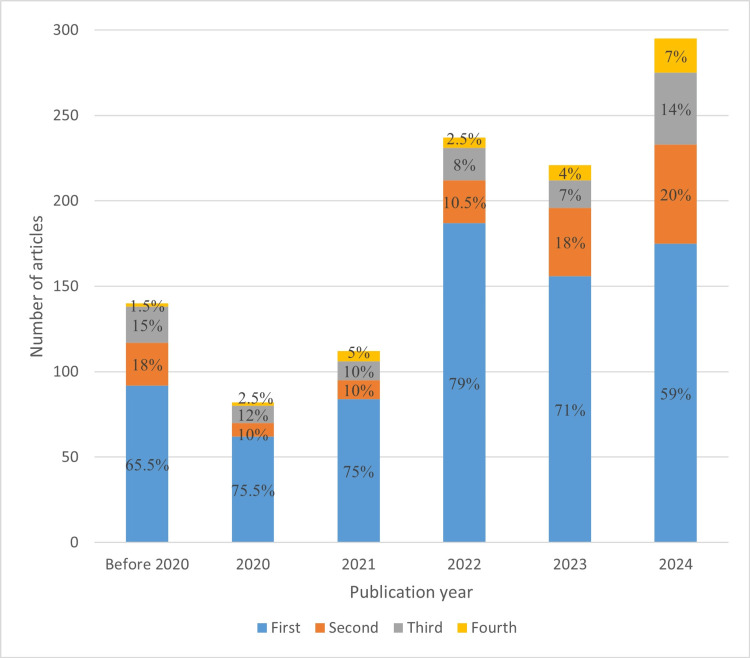
Distribution of studies for each treatment stage across different publication year groups First stage, examination and diagnosis; second stage, treatment planning; third stage, operative phase; fourth stage, follow-up and maintenance

Table 2 provides the number of original studies categorized by dental disciplines and stages of dental treatment. Figure 6 and Figure 7 present the distribution of studies for each discipline and treatment stage across different publication year groups. Orthodontics emerged as the most studied discipline, followed by oral and maxillofacial radiology, research and education, and operative dentistry. The majority of studies applied AI in the first stage of dental treatment.

**Table 2:** Number of original studies in each discipline AI: artificial intelligence

	Applications in dental disciplines												
Stages of AI application in dentistry	Oral medicine/pathology	Periodontics	Endodontics	Orthodontics	Pediatric dentistry	Oral and maxillofacial surgery	Implantology	Prosthodontics	Operative dentistry	Oral and maxillofacial radiology	Forensic dentistry	Oral public health	Research and education
Examination and diagnosis	87	64	46	97	37	36	7	45	109	147	77	36	134
Treatment planning, prognosis, and prediction	14	12	6	44	9	24	19	26	13				
Operation (intervention)	0	0	11	26	2	5	16	49	8				
Follow-up and maintenance	2	6	8	6	1	5	10	5	2				

The results of trend analysis and forecasted values for 2025 are presented in Table 3. After 2022, there was a significant increase in research and education studies, with the highest number of studies in 2025 forecasted at 72 studies. Regarding treatment stages, the second and fourth stages gained increased attention after 2021, although the fourth stage received the least attention overall. Notably, the second and third stages exhibited high relative popularity in the before 2020 and 2024 groups. While the first stage remained the most prevalent across all year groups, its relative prominence is declining, with the highest increase in 2025 forecasted for the second stage, accounting for 24% of studies.

**Table 3:** Estimated values for the whole of 2024 and method of forecasting and forecasted values for 2025 DES: Double Exponential Smoothing

	Estimated for 2024	Forecasting method	Forecasted for 2025
Dental discipline			
Orthodontics	57	LINEAR Function	65
Research and education	58	Log-ETS Function	72
Stage of treatment			
First	59%	LINEAR Function	58%
Second	20%	Log-ETS Function	24%
Data modality			
Text	183	Log-ETS Function	249
Radiography	183	LINEAR Function	209
Others	41	Holt’s DES	122
Subsets of artificial intelligence			
Deep learning	212	ETS Function	216
Natural language processing	38	Log-ETS Function	71
Hybrid models	32	ETS Function	36
Traditional machine learning algorithm			
Random forest	33	LINEAR Function	38
Model of deep learning			
Convolutional neural networks	157	Log-ETS Function	199
Hybrid models	46	LINEAR Function	58
Tasks of artificial intelligence			
Classification	141	LINEAR Function	165
Detection	98	ETS Function	113
Segmentation	93	LINEAR Function	114
Generation	38	Log-ETS Function	49
Data integration	41	ETS Function	49
Decision support	58	LINEAR Function	61

Review Studies

Table 4 summarizes the number of review studies. Approximately 77% (188 out of 244) of these articles were general reviews, which did not focus on specific AI applications within particular disciplines. The remaining reviews systematically evaluated AI applications in specific disciplines. Comparing the statistics of primary materials in Table 2 with the distribution of specific review studies in Table 4 highlights potential areas for future systematic reviews.

**Table 4:** Number of review studies in each discipline

	Applications in dental disciplines												
	Oral medicine/pathology	Periodontics	Endodontics	Orthodontics	Pediatric dentistry	Oral and maxillofacial surgery	Implantology	Prosthodontics	Operative dentistry	Oral and maxillofacial radiology	Forensic dentistry	Oral public health	Research and Education
Systematic review in specific discipline	3 (two stage 1 and one stage 2)	6 (four stage 1 and two stage 2)	3 (multi-stage)	10 (five stage 1, two stage 2, and three multi-stage)	3 (one stage 1 and two multi-stage)	2 (one stage 1 and one stage 2)	5 (one stage 2, three stage 3, and one multi-stage)	4 (one stage 2, one stage 3, and two multi-stage)	6 (stage 1)	11	1	1	1
General	188												

The highest priority for new systematic reviews lies in the field of AI applications in research and education. The second priority should address the first-stage studies within prosthodontics and endodontics. Conversely, conducting additional general review studies appears to have limited value. Furthermore, disciplines such as orthodontics and radiology already have satisfactory systematic reviews.

Data Modality

**Figure 8:**
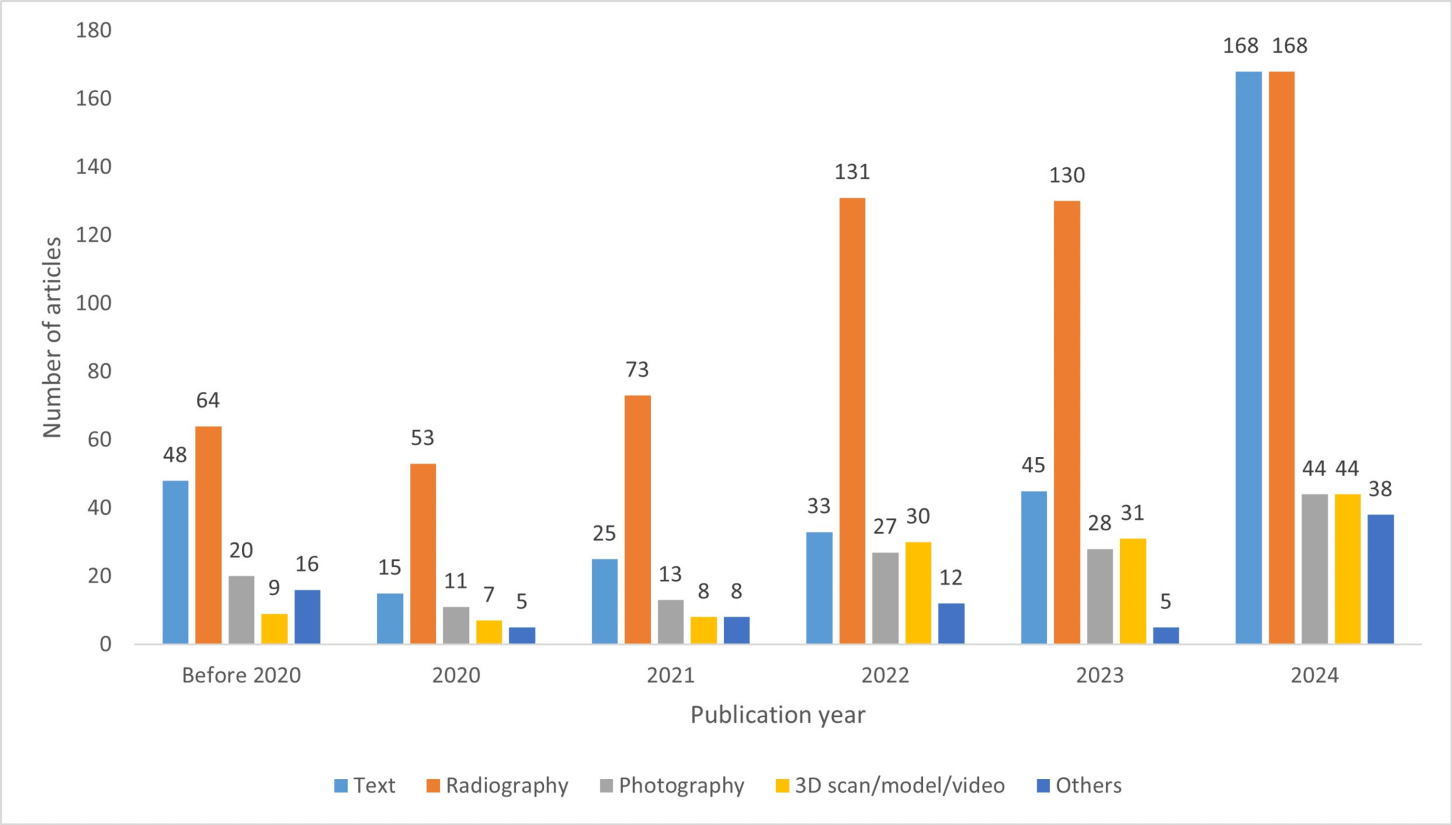
Distribution of data modalities across the publication year groups

The distribution of data modalities across the six publication year groups is displayed in Figure 8. Radiographic materials emerged as the most frequently used data modality across all groups. While the quantity of all data modalities increased over time, the most significant growth was observed in textual materials, with the highest forecasted value for 2025 at 249 studies. The “others” category, which includes physiological signals, ultrasound and infrared images, sensor data, and new other modalities, showed a noticeable surge in 2024.

Subsets of Artificial Intelligence

**Figure 9:**
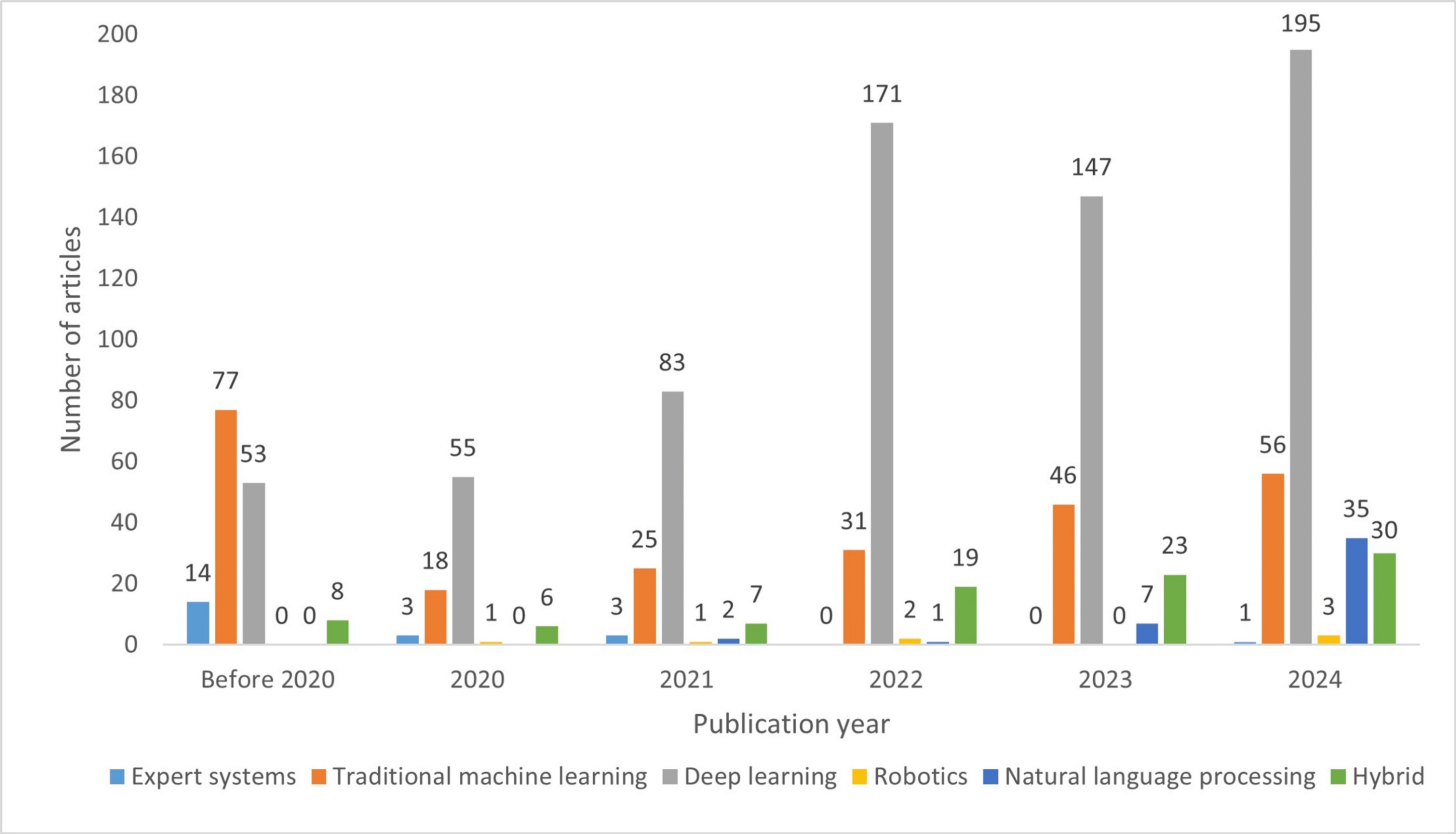
Distribution of artificial intelligence subsets across the publication year groups

Figure 9 illustrates the trends in utilizing different AI subsets over the six publication year groups. TML was the most frequent subset before 2020. However, in subsequent years, DL became the most dominant AI type. The recent surge in the adoption of hybrid models and, more notably, NLP applications (71 studies in 2025) is evident. The only subset showing a declining trend is expert systems, while robotics incorporating true AI continues to grow.

Algorithms and Models

**Figure 10:**
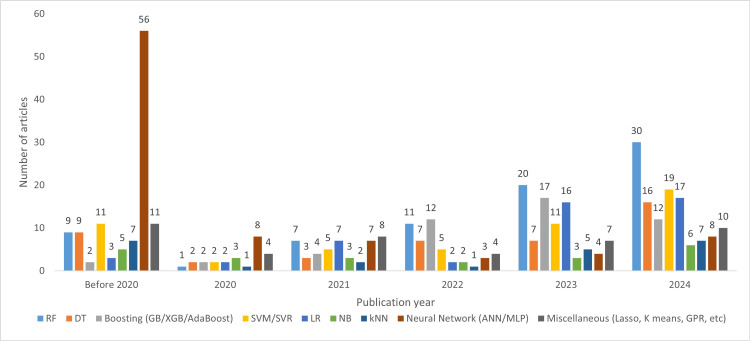
Distribution of traditional machine learning algorithms across publication year groups RF, random forest; DT, decision tree; GB, gradient boosting; XGB, extreme gradient boosting; SVM, support vector machine; SVR, support vector regression; LR, logistic regression; NB, naive Bayes; kNN, k-nearest neighbors; ANN, artificial neural network; MLP, multilayer perceptron; GPR, Gaussian process regression

Figure 10 depicts the trends in commonly used TML algorithms in dentistry. Neural networks experienced a significant decline after 2020, while the overall use of other algorithms, particularly random forests, has steadily increased in recent years, with the highest forecasted number of studies in 2025 at 38.

**Figure 11:**
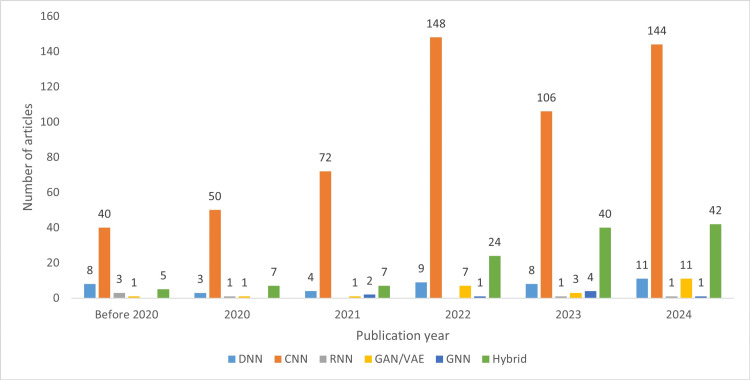
Distribution of deep learning models across publication year groups DNN, deep neural network; CNN, convolutional neural network; RNN, recurrent neural network; GAN, generative adversarial network; VAE, variational autoencoder; GNN, graph neural network

**Figure 12:**
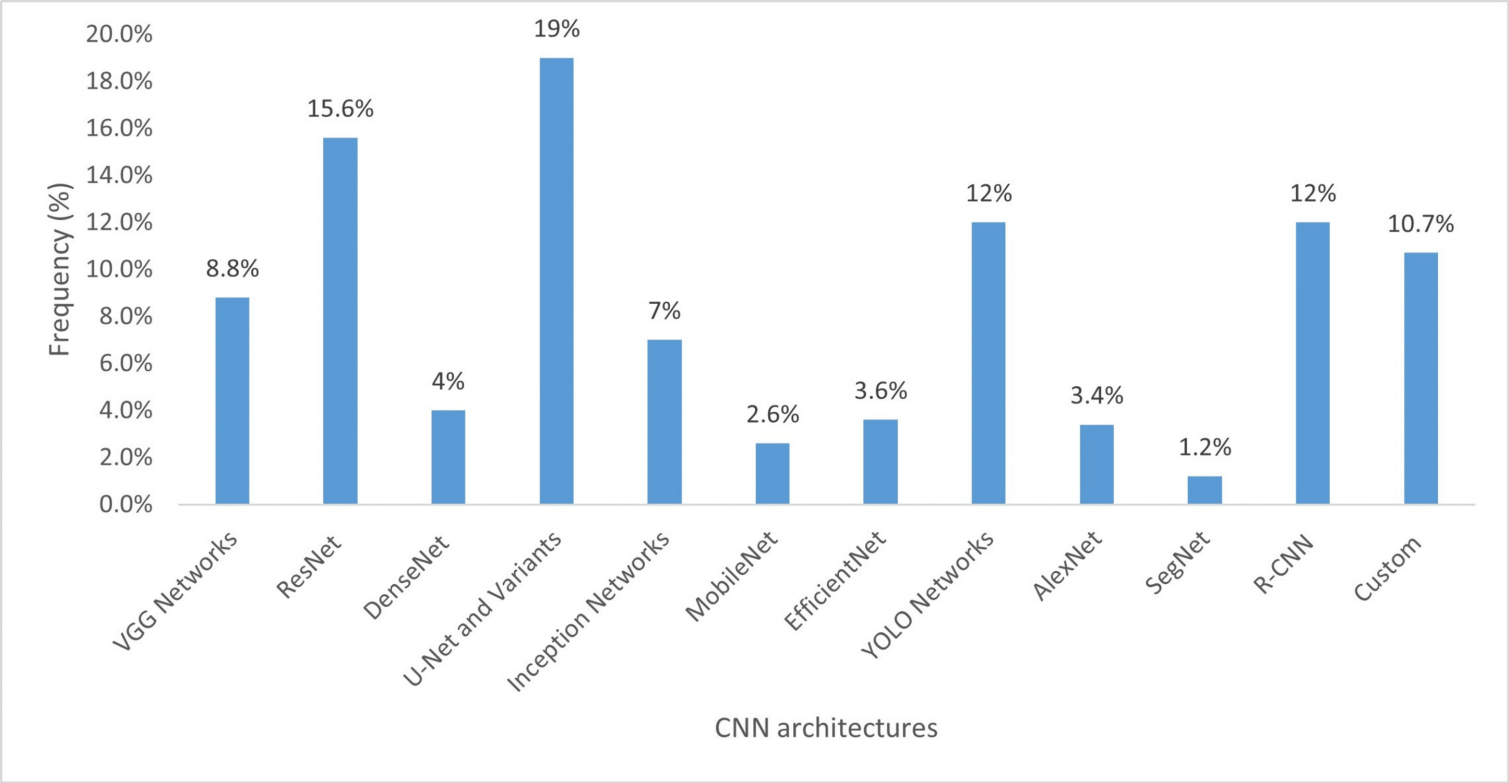
Distribution of different convolutional neural network architectures Custom: BasicVSR, ConvNext, DentNet, DetectNet, RetinaNet, etc. VGG, Visual Geometry Group; ResNet, residual networks; DenseNet, densely connected convolutional networks; YOLO, You Only Look Once; R-CNN, region-based convolutional neural network

Figure 11 illustrates the trends in DL models within dentistry. Convolutional neural networks (CNNs) continue to be the most widely used DL model in the field. Since 2021, there has been significant growth in the adoption of generative models, including generative adversarial networks (GANs) and variational autoencoders (VAEs), with the highest increase observed in hybrid deep learning models, which are forecasted to reach 58 studies by 2025. While recurrent neural networks (RNNs) are increasingly utilized as components of hybrid DL models, their independent application in dentistry remains uncommon. The use of graph neural networks (GNNs) emerged in late 2021, but their applications in dentistry are still relatively limited. Figure 12 provides the distribution of different CNN architectures, with U-Net and residual networks (ResNet) being the most employed.

Artificial Intelligence Tasks in Dentistry

**Figure 13:**
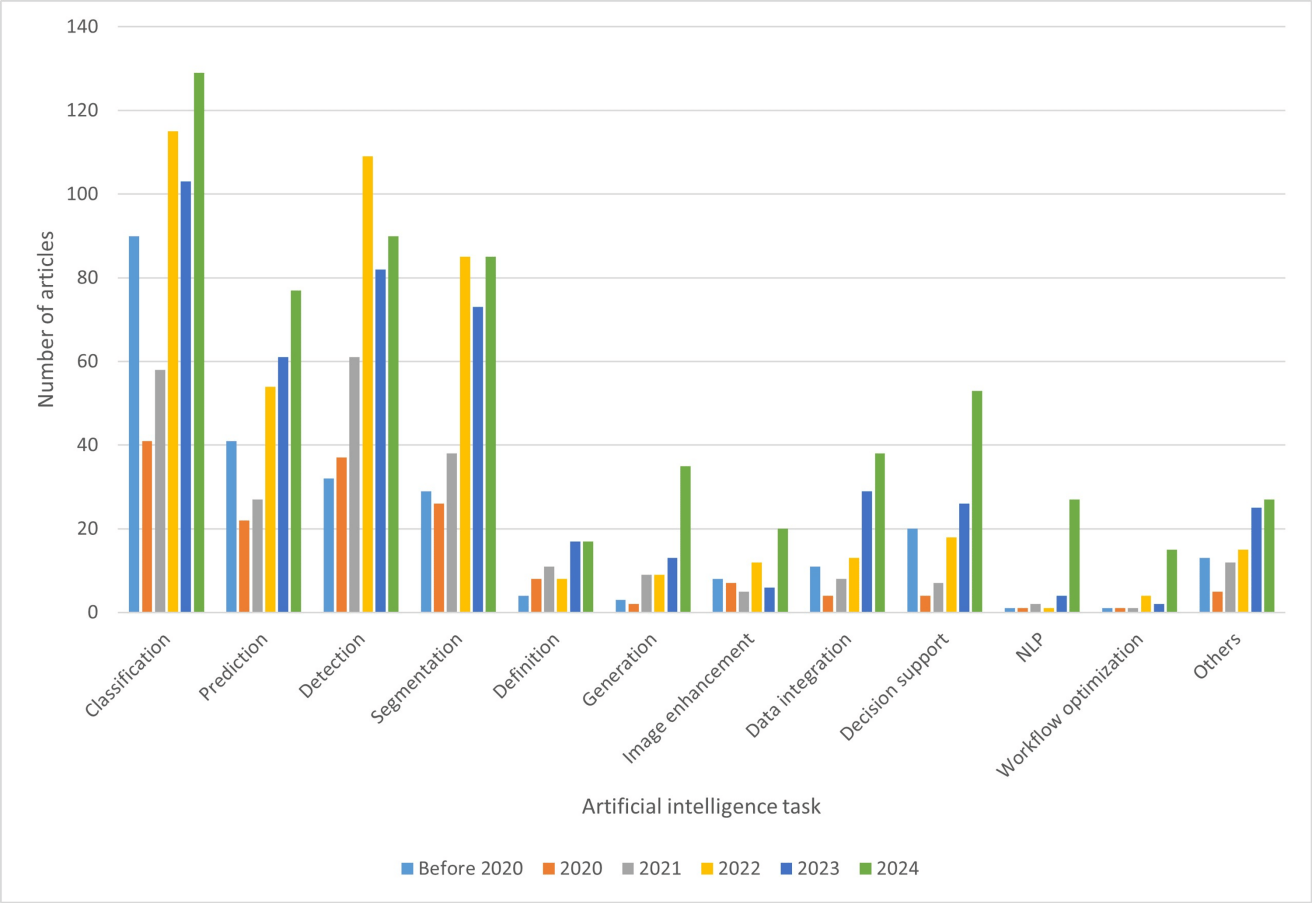
Distribution of artificial intelligence tasks across publication year groups NLP: natural language processing

AI applications in dentistry encompass a wide range of tasks. This study identified at least 16 distinct tasks performed by AI in dentistry, with classification, detection, and segmentation being the most popular. The tasks and their definitions are mentioned in Table 5. Figure 13 illustrates trends in tasks that have undergone significant changes, with notable increases observed in generation, data integration, and decision support. While NLP, image enhancement, and workflow optimization demonstrated considerable growth, their limited numbers precluded comprehensive analysis and forecasting for 2025.

**Table 5:** Artificial intelligence tasks and their definition (application) in dentistry

Task	Definition (application)
Classification	Categorizing data (healthy versus diseased)
Detection	Identifying and localizing entities (caries, lesions, and crowns)
Segmentation	Dividing images into parts (caries extension, restoration margins, and tooth and bone segmentation)
Prediction	Forecasting outcomes (prognosis and success rates)
Definition	Standardizing terms (disease names and tooth numbering)
Generation	Creating or simulating data (creating new text or image and designing restoration)
Image enhancement	Improving image quality (radiographs)
Data integration and management	Registration and combining different datasets (clinical-paraclinical or radiography-3D scan)
Decision support	Recommending treatment plans or identifying anomalies
Natural language processing	Analyzing textual data (electronic health records)
Workflow optimization	Automating tasks (scheduling)
Others	Includes robotics, quantification (measurement), data analysis, and monitoring

Classification of Utilized Artificial Intelligence in Dentistry

**Figure 14:**
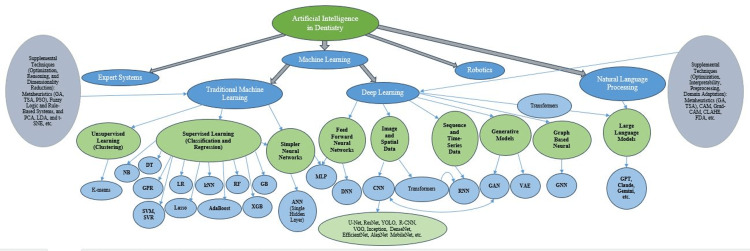
Classification of artificial intelligence utilized in dentistry GA, genetic algorithm; TSA, tabu search algorithm; PSO, particle swarm optimization; PCA, principal component analysis; LDA, linear discriminant analysis; t-SNE, t-distributed stochastic neighbor embedding; RF, random forest; DT, decision tree; GB, gradient boosting; XGB, extreme gradient boosting; SVM, support vector machine; SVR, support vector regression; LR, logistic regression; NB, naive Bayes; kNN, k-nearest neighbors; ANN, artificial neural network; MLP, multilayer perceptron; GPR, Gaussian process regression; DNN, deep neural network; CNN, convolutional neural network; RNN, recurrent neural network; GAN, generative adversarial network; VAE, variational autoencoder; GNN, graph neural network; VGG, Visual Geometry Group; ResNet, residual networks; DenseNet, densely connected convolutional networks; YOLO, You Only Look Once; R-CNN, region-based convolutional neural network; GPT, generative pre-trained transformer; CAM, class activation mapping; CLAHE, contrast limited adaptive histogram equalization; FDA, factorial discriminant analysis

Based on the AI systems employed in dentistry and their classifications in computer science, a comprehensive framework is proposed in Figure 14 [4,20]. AI systems in dentistry can be categorized into five subsets: expert systems, TML, DL, robotics, and NLP.

Expert systems are predefined rule-based AI systems designed to simulate the decision-making processes of a human expert in dentistry, which are the least used in dentistry [27]. Robotics was previously underutilized; however, its applications incorporating true AI are increasing. TML algorithms include supervised models (e.g., random forest {RF}, decision tree {DT}, and super vector machines {SVM}) and unsupervised models. Basic neural networks and multilayer perceptron are also part of this subset.

DL is the largest subset, encompassing feedforward neural networks (deep neural network {DNN} with multiple hidden layers) and models for image and spatial data (CNN and transformers), sequence and time-series data (RNN), generation (GAN and VAE), and graph-based data (GNN). CNNs, particularly architectures such as U-Net and ResNet, are the most widely used. Hybrid DL models that integrate different models are growing in popularity. The last subset is NLP, which is an independent subset but built on DL (especially transformers) that has seen rapid growth [24,25]. Its applications in dentistry include large language models (LLMs), such as ChatGPT and Gemini, which are particularly beneficial for analyzing textual data and supporting novel research initiatives.

Discussion

This study aimed to evaluate trends in the data modalities, subsets, models, and tasks of AI employed in various dental disciplines and treatment stages and to suggest novel research directions based on current preferences for AI applications in the field. Given the observed trends, future research should focus on the research and education discipline and the treatment planning stage of dental treatments and NLP methods and hybrid models utilizing textual data and new modalities. Furthermore, this study provided a comprehensive list of frequently used AI systems in dentistry, proposed a classification for these systems, and briefly explained their applications.

The application of AI in dentistry has gained increasing attention, reflected in a notable rise in publications. However, the strict inclusion criteria of focusing on true AI applications excluded 119 articles, primarily robotics studies. While robotics has long been a research focus, AI integration within robotics remains relatively new in dentistry.

Included studies were categorized as primary studies, proof-of-concept papers, or reviews. The recent surge in primary and review studies suggests growing familiarity with AI among dental researchers, shifting from early proof-of-concept work toward model evaluation and comparison [17]. This trend aligns with the increasing presence of dentistry-related keywords and the greater engagement of dental journals with AI research. By reporting frequent keywords, sources, and authors, this study provides valuable insights for emerging researchers and guidance on potential future research directions [17].

Trends in Disciplines and Stages of Dental Treatment

Based on trends in the distribution of AI applications, future research should prioritize the research and education discipline, as well as the treatment planning stages of dental treatment. One potential driver of these trends is the development of hybrid AI models, particularly those incorporating natural language processing (NLP). While earlier studies focused on TML and DL for general tasks, such as detection, classification, and the segmentation of radiographic images or dental scans, recent advancements in large language models (LLMs) such as ChatGPT and Gemini have expanded the potential of NLP for analyzing textual data [24]. Additionally, improved DL architectures, such as those combining convolutional neural networks with generative adversarial networks, are enhancing the capabilities of AI in generation tasks [12].

AI research in dentistry is predominantly focused on diagnostic applications. However, recent years have seen growing attention to the other three stages of dental treatment. Interestingly, stages 2 and 3 gained more prominence before 2020 and again in 2024. Prior to 2020, TML models were widely employed, with predictive models offering significant utility. With the advancement of DL, diagnostic applications became the primary focus. By 2024, the development of sophisticated DL and hybrid models expanded AI’s capabilities to include data integration, workflow optimization, and generative tasks, enhancing its impact on treatment planning and operative stages [23,26].

Researchers seeking novel ideas may find opportunities to explore AI applications in the second stage of dental treatment. In this study, both “prediction and prognosis” and “treatment planning” were classified as part of the second stage of dental treatment due to their interdependence. Most AI applications in this stage were employed for predictive tasks and orthodontic treatment planning. Future research could focus on developing AI tools for treatment planning in other disciplines, a promising area given the potential of LLMs and hybrid models.

Focus on Review Studies

The increasing interest in review studies among researchers was evident. While general review studies on AI applications in dentistry are numerous, systematic reviews addressing specific disciplines, stages, or AI models need more attention. Notably, the research and education discipline, which has seen a surge in AI-related studies, lacks comprehensive systematic reviews, representing a significant knowledge gap. Similarly, systematic reviews focusing on the diagnostic stages of prosthodontics and endodontics offer a high potential for novelty. Conversely, disciplines such as orthodontics and radiology already have multiple systematic reviews, and additional general reviews are unlikely to provide substantial new insights.

Trends in Different Artificial Intelligence Subsets, Models, Data Modalities, and Tasks

The increasing prevalence of textual data modalities is attributed to advancements in NLP models, useful in research and education [23-25]. Additionally, the growing sophistication of AI systems is enabling the integration of novel data types, such as sensor and signal data, which could lead to the development of integrated models capable of performing complex tasks [28].

The growing popularity of DL in dental applications is evident, although TML remains widely employed. The use of neural network applications within TML algorithms has declined. A possible reason for this reduction is the increasing complexity of modern neural networks, which now classify them as part of DL models [29]. Beyond the use of unsupervised and supervised algorithms in dentistry, there are supplemental algorithms that enhance the performance of TML models. As presented in Figure 5, these algorithms, which focus on optimization, reasoning, and dimensionality reduction, are not inherently considered AI. Similarly, for DL-based models, there are adjunctive algorithms, also non-AI by definition, that improve optimization, interpretation, preprocessing, and domain adaptation [10,21].

Hybrid AI models, integrating different algorithms and approaches, are extending the applicability of AI in dentistry. Common examples include combining different models of DL together, incorporating TML with DL to improve accuracy, and integrating ML with robotics to enhance reasoning and learning capabilities [26,29]. NLP, which is inherently a hybrid AI, is treated as a separate subset due to its distinct functionalities [24,25].

Classification of Utilized Artificial Intelligence in Dentistry

AI has rapidly transformed dentistry by enhancing diagnostics, treatment planning, operations, and follow-up care [8-12]. The following sections discuss the dentistry-related applications of the mentioned AI models and algorithms in the proposed classification.

Expert systems: Previously used primarily for decision support tasks, expert systems have seen a decline in popularity due to advancements in other AI subsets [9,27].

TML: Supervised algorithms such as RF, DT, and boosting models are employed for predicting caries risk, classifying oral lesions, and forecasting treatment success. SVM is particularly effective in classifying oral lesions from radiographic or histological images, and logistic regression supports the prediction of treatment outcomes. K-means, as an unsupervised method, are used for patient clustering to enable personalized treatment planning and oral public health interventions [10,21].

DNN: DNN is highly effective in analyzing clinical data for various applications, including disease identification, decision support, and predictive tasks such as caries risk assessment, periodontal disease prediction, and estimating the costs of dental procedures [8,21].

CNN: CNNs are widely applied in analyzing radiographic images for detecting caries, fractures, cysts, and bone density issues. CNNs also support cephalometric analysis, tooth segmentation on images and 3D scans, implant placement planning, and oral cancer detection using clinical or histopathological images [8,21].

RNN: RNNs analyze sequence and time-series data, assisting in tracking temporomandibular joint movements and evaluating abnormal chewing functions [10,30].

GAN and VAE: GAN and VAE generate 3D models for orthodontics, prosthodontics, and surgical planning and enhance dental image quality for improved diagnostics. These models also create photorealistic simulations of treatment outcomes, such as smile design, and virtual reality environments for education and training [8,31].

GNN: GNNs can be applied in predicting the next disease, periodontal mapping, modeling disease progression, analyzing complex relationships between the teeth and supporting structures for orthodontic and surgical planning, mapping occlusal forces, and simulating implant-supported restorations [32].

Robotics: While the integration of true AI in robotics is relatively new, it holds promise for improving dental procedures, reducing patient recovery times, and enhancing treatment precision. Applications include robotic systems for implant placement, orthodontic wire bending, minimally invasive surgeries, and micro-robotics in endodontics [33].

NLP: These models are transforming patient-clinician interactions and administrative tasks. They support automated charting, improve record-keeping, and extract insights from unstructured electronic health records (EHRs). NLP technologies, using virtual assistants and chatbots, may facilitate automated scheduling, provide instructions, and enhance service quality monitoring [24,25].

Hybrid AI models: Hybrid models combine multiple AI subsets, offering integrated solutions for complex tasks. For example, hybrid models in prosthodontics integrate CNNs and GANs for designing prostheses while ensuring material selection and functional success. These models also enable interdisciplinary approaches, such as combining textual, radiographic, and histological data to deliver comprehensive diagnostic and treatment plans [9,23,28].

Limitations and Recommendations

This study did not examine the structural intricacies of AI models but focused on trends in data modalities and AI applications across dental treatment stages. While many studies utilized radiographic data, they were classified under radiology only when AI was explicitly applied to image interpretation or enhancement [20]. Studies using radiographic data for tasks such as caries detection or landmark identification were assigned to their respective disciplines.

A limitation of this study was the exclusion of traditional robotics research that did not meet the criteria for “true AI.” Although this ensured consistency with the study’s focus, it omitted a substantial body of robotics literature. However, the increasing integration of AI into robotic systems suggests a growing role for robotics in the operative stage of dental treatment. Another limitation of this study was the search date, which included only publications up to the end of November. To address this, an estimation of the annual publication count was performed to approximate the full 12-month statistics of 2024.

Another limitation of this study is the restriction of the literature search to the Web of Science Core Collection. This decision was made based on the database’s high indexing standards, compatibility with bibliometric analysis tools, and structured metadata that support accurate co-authorship analyses. Moreover, this approach aligns with established bibliometric guidelines and previous studies [15-19]. Nonetheless, future research may benefit from incorporating additional databases, such as Scopus or PubMed, to enhance the comprehensiveness and validation of the findings. Furthermore, the decision to group all studies published before 2020 into a single category was made because the number of annual publications during that period was limited. Disaggregating them by year would have resulted in insufficient data points, reducing the reliability of trend analyses.

This study serves as a roadmap for future AI-driven innovations in dentistry by offering a structured overview of previous and current applications, highlighting emerging trends, and revealing research gaps. The introduced classification framework can foster consistency in AI research across dental domains, supporting clearer comparisons and collaboration. By emphasizing the growing importance of hybrid models, NLP, and underrepresented treatment stages such as treatment planning, the study encourages the development of next-generation AI tools that enhance clinical decision-making and dental education.

## Conclusions

This study employs a novel evaluative approach to AI applications in dentistry and identifies key areas for future AI research in the field, particularly original studies in research and education and the treatment planning stage. Systematic reviews on AI applications in research and education and the diagnostic stages of prosthodontics and endodontics offer high potential for novelty, while general reviews are less likely to make significant contributions.

Future research should prioritize NLP applications, advanced deep learning models, hybrid AI systems, and emerging data modalities such as sensor data and signal processing. AI tasks, including data integration, generation, and decision support, are rapidly evolving, highlighting them as promising areas for further investigation.

## References

[1] Janiesch C, Zschech P, Heinrich K (2021). Machine learning and deep learning. Electron Mark.

[2] Wang P (2019). On defining artificial intelligence. J Artif Gen Intell.

[3] LeCun Y, Bengio Y, Hinton G (2015). Deep learning. Nature.

[4] Mukhamediev RI, Popova Y, Kuchin Y (2022). Review of artificial intelligence and machine learning technologies: classification, restrictions, opportunities and challenges. Mathematics.

[5] Molenaar I (2022). Towards hybrid human‐AI learning technologies. Eur J Educ.

[6] Strzelecki M, Badura P (2022). Machine learning for biomedical application. Appl Sci.

[7] Sengar SS, Hasan AB, Kumar S, Carroll F (2024). Generative artificial intelligence: a systematic review and applications. Multimed Tools Appl.

[8] Ding H, Wu J, Zhao W, Matinlinna JP, Burrow MF, Tsoi JK (2023). Artificial intelligence in dentistry-a review. Front Dent Med.

[9] Schwendicke F, Samek W, Krois J (2020). Artificial intelligence in dentistry: chances and challenges. J Dent Res.

[10] Shan T, Tay FR, Gu L (2021). Application of artificial intelligence in dentistry. J Dent Res.

[11] Agrawal P, Nikhade P (2022). Artificial intelligence in dentistry: past, present, and future. Cureus.

[12] Al-Haddad AA, Al-Haddad LA, Al-Haddad SA, Jaber AA, Khan ZH, Rehman HZ (2024). Towards dental diagnostic systems: synergizing wavelet transform with generative adversarial networks for enhanced image data fusion. Comput Biol Med.

[13] Khanagar SB, Al-Ehaideb A, Maganur PC (2021). Developments, application, and performance of artificial intelligence in dentistry - a systematic review. J Dent Sci.

[14] Xiang B, Lu J, Yu J (2024). Evaluating tooth segmentation accuracy and time efficiency in CBCT images using artificial intelligence: a systematic review and meta-analysis. J Dent.

[15] Montazeri A, Mohammadi S, M Hesari P, Ghaemi M, Riazi H, Sheikhi-Mobarakeh Z (2023). Preliminary guideline for reporting bibliometric reviews of the biomedical literature (BIBLIO): a minimum requirements. Syst Rev.

[16] Öztürk O, Kocaman R, Kanbach DK (2024). How to design bibliometric research: an overview and a framework proposal. Rev Manag Sci.

[17] Ellegaard O (2018). The application of bibliometric analysis: disciplinary and user aspects. Scientometrics.

[18] Zatt FP, Rocha AO, Anjos LM, Caldas RA, Cardoso M, Rabelo GD (2024). Artificial intelligence applications in dentistry: a bibliometric review with an emphasis on computational research trends within the field. J Am Dent Assoc.

[19] Xie B, Xu D, Zou XQ, Lu MJ, Peng XL, Wen XJ (2024). Artificial intelligence in dentistry: a bibliometric analysis from 2000 to 2023. J Dent Sci.

[20] Liu R, Ramli AA, Zhang H, Henricson E, Liu X (2022). An overview of human activity recognition using wearable sensors: healthcare and artificial intelligence. Internet of Things - ICIOT 2021.

[21] Carrillo-Perez F, Pecho OE, Morales JC (2022). Applications of artificial intelligence in dentistry: a comprehensive review. J Esthet Restor Dent.

[22] Stefanac S, Nesbit S (2024). Diagnosis and treatment planning in dentistry: diagnosis and treatment planning in dentistry. https://books.google.ca/books?hl=en&lr=&id=HcCrEAAAQBAJ&oi=fnd&pg=PP1&dq=Diagnosis+and+Treatment+Planning+in+Dentistry:+Diagnosis+and+Treatment+Planning+in+Dentistry&ots=audloHtUwm&sig=NbdXHOXqFFeoXqgX2h9tC9Q19ok&redir_esc=y#v=onepage&q=Diagnosis%20and%20Treatment%20Planning%20in%20Dentistry%3A%20Diagnosis%20and%20Treatment%20Planning%20in%20Dentistry&f=false.

[23] Feher B, Tussie C, Giannobile WV (2024). Applied artificial intelligence in dentistry: emerging data modalities and modeling approaches. Front Artif Intell.

[24] Houssein EH, Mohamed RE, Ali AA (2021). Machine learning techniques for biomedical natural language processing: a comprehensive review. IEEE Access.

[25] Büttner M, Leser U, Schneider L, Schwendicke F (2024). Natural language processing: chances and challenges in dentistry. J Dent.

[26] Dixit S, Kumar A, Srinivasan K (2023). A current review of machine learning and deep learning models in oral cancer diagnosis: recent technologies, open challenges, and future research directions. Diagnostics (Basel).

[27] Saibene A, Assale M, Giltri M (2021). Expert systems: definitions, advantages and issues in medical field applications. Expert Syst Appl.

[28] Acosta JN, Falcone GJ, Rajpurkar P, Topol EJ (2022). Multimodal biomedical AI. Nat Med.

[29] Manisha Manisha, Dhull SK, Singh KK (2020). ECG beat classifiers: a journey from ANN to DNN. Procedia Comput Sci.

[30] Kumari AR, Rao SN, Reddy PR (2022). Design of hybrid dental caries segmentation and caries detection with meta-heuristic-based ResneXt-RNN. Biomed Signal Process Control.

[31] Alamir M, Alghamdi M (2022). The role of generative adversarial network in medical image analysis: an in-depth survey. ACM Comput Surv.

[32] Tseng YC, Li WC, Peng WC, Hung CC (2024). Predicting the next diseases using graph neural networks on administrative medical datasets. J Inf Sci Eng.

[33] Xia Z, Ahmad F, Deng H, Jiang L, Qin W, Zhao Q, Xiong J (2024). Robotics application in dentistry: a review. IEEE Trans Med Robot Bionics.

